# Mothers’ views about sexual health education for their adolescent daughters: a qualitative study

**DOI:** 10.1186/s12978-017-0291-8

**Published:** 2017-02-10

**Authors:** Mohsen Shams, Sa’adat Parhizkar, Ali Mousavizadeh, Masoumeh Majdpour

**Affiliations:** 1 Health Education, Social Determinants of Health Research Center, Yasuj University of Medical Sciences, Yasuj, Iran; 2 Reproductive Health, Social Determinants of Health Research Center, Yasuj University of Medical Sciences, Yasuj, Iran; 3 Epidemiology, School of Public Health, Yasuj University of Medical Sciences, Yasuj, Iran; 4 M.Sc. in Health Education and Promotion, School of Public Health, Yasuj University of Medical Sciences, Yasuj, Iran

**Keywords:** Sexual health, Education, Adolescents, Qualitative study

## Abstract

**Background:**

Given that mothers play a role in the sexual education of their daughters, it is important to understand their views of sexual health and related programs. This study was aimed at exploring mothers’ perspectives regarding sexual health education for their adolescent daughters in Mahshahr, Iran.

**Methods:**

In this qualitative study, in-depth interviews with ten key informants and five focus group discussions involving 28 mothers with daughters aged 12–18 were conducted. All the discussions were audio-recorded and later transcribed. The data were classified, after which the main themes and sub-themes were manually extracted and analyzed.

**Results:**

The five main themes determined were: the necessity of sexual health education for adolescent girls, the sources of information that mothers use, barriers to sexual health education, the need to empower mothers to provide sexual education to their daughters, and recommendations for developing special training programs for mothers. Most participants believed in limiting sexual health education for adolescent girls; nevertheless, they stated that trained mothers were best equipped to educate their daughters. The major barriers identified by the mothers were their own insufficient knowledge about sexual issues, embarrassment surrounding discussions of this issue with their daughters, fear of the arrogance and curiosity of girls, and a lack of skills for effective communication.

**Conclusion:**

The results showed that empowering mothers to provide sexual health education is important. Tailored educational programs, based on mothers’ views, should be developed and implemented.

## Plain English summary

Mothers play an important role in educating adolescent girls regarding sexual health. Understanding their views about sexual education can therefore enable the design of effective interventions. This qualitative research, conducted in Mahshahr, Iran, explored and analyzed the views of mothers with daughters aged 12–18 and the views of other key informants. Data were collected through focus group discussions and interviews. The participants discussed the need for sexual health education for adolescent girls, the resources that mothers use to derive information on sexual health education, barriers to sexual health education, the need to empower mothers so that they can educate their daughters regarding sexual health, and recommendations for developing special training programs for mothers.

## Background

More than 50% of the world’s population begins their sexual activity in their youth [1]. Empowering adolescents to improve their sexual health can help them effectively deal with related problems, such as infertility, sexually transmitted infections, and high-risk sexual behaviors; it can also encourage the younger generation to rationally approach and take responsibility for sexual relations [2]. Sexual health education programs are necessary for individuals who have not yet begun engaging in sexual activities and those who are already sexually active [5]. Sexual health education is “a lifelong process of acquiring information and forming attitudes, beliefs, and values about such important topics as identity, relationships, and intimacy” [3]. Despite its importance for adolescents, sexual health education is a complex endeavor [4].

According to the policies of Iranian Ministry of Health, changes in the social and cultural structure of the population have made sexual health education an important health priority [5]. Although Iranians engage in sexual activities at a young age, often before marriage, no formal systematic sexual health education program is available to adolescents [4]. A major driver of opposition to the provision of sexual health education in the country is due to misperceptions of these programs [6]. Consequently, adolescents are compelled to search for sexual health information on the Internet or through peers. This approach may lead to the sharing of false or exaggerated information. Some teachers, principals, and education officials remain unconvinced of the need to educate adolescents about sexual health, and thus oppose such programs [6].

Methods for sexual health education are often based on societal beliefs and values [7]. The family, as an individual’s first social entity, plays a key role in socialization [8]. In families, the role of mothers is more pronounced than those of other members, and adolescents learn healthy behaviors through maternal guidance [9]. Some mothers, however, hold negative attitudes toward sexual health education for adolescents, and only a small percentage can answer their children’s questions about sexual health [10]. Some studies have shown that parents provide consent to others who are willing to educate their teens [11]. As stated by certain adolescents, the lack of awareness and communication skills among parents is a principal reason why they do not discuss sexual issues with their parents [12]. As a fact, girls and boys must be learnt to deal with their sexual issues, and therefore, they search for easier ways to access to accurate information. In a study conducted in Tehran, mothers stated that sexual education should be provided to girls and parents, especially mothers [13]. Educated women serve to communicate such information to spouses, children, co-workers, and peers [14]. Most school-based health teachers believe that sexual health education should be initiated by parents; therefore, family programs should prepare them to address their children’s sex-related questions [12]. Motivated by the important role of parents as participants in the sexual health education, some research has focused on empowerment programs for families in an effort to improve sexual health [15]. This study explored the perspectives of mothers regarding sexual health education for their adolescent girls in Mahshahr City, Iran.

## Methods

This qualitative study was conducted in June to September 2015. The target group for this study was adolescent girls (12–18 years), their mothers, and key informants in the area of adolescent sexual health. Data were collected by interview and semi-structured focused group discussion. Interviews with key informants included one with a high school principal, three with school counselors, and three with local midwives; the midwives were also mothers to adolescent girls. For each focus group discussion, 12 mothers were invited, resulting in a total of 28 participants in five focus groups. Each interview and focus group lasted one to one-and-half hours. Informed consent was obtained from the participants prior to discussions. Trained interviewers and focus group coordinators led all interviews and group discussions for collecting relevant data. MM (the corresponding author) was present at all focus group sessions. Discussions and interview questions were designed as follows:

1. What are your ideas about sexual health education for adolescent girls?
2. If you think sexual health education is necessary, how must this education be provided?
3. What barriers to the provision of sexual health education to adolescent girls have you encountered?
4. Do mothers need to be trained to educate adolescent girls about sexual health? How should such training be delivered?

All discussions were transcribed verbatim and manually analyzed by an experience qualitative researcher in the field of public health. The original transcripts were copied, cut, pasted, and categorized using color-coding. To ensure the context of responses was appropriately reflected, all transcripts were repeatedly reviewed. The transcripts were analyzed for descriptive summaries and comparisons between groups. The researcher, who did the first analysis, reviewed the summaries to confirm the analysis. Credibility of results was improved by ensuring diversity of participants in terms of age, ethnicity, educational level, socio-economic status, employment, and education. The Research Ethics Committee of Yasuj University of Medical Sciences in Iran approved this study (approval code: 930120308), and the research was registered in Iranian Registry of Clinical Trials (IRCT) by the number of IRCT2015060622575N1.

## Results

Table 1 shows the demographic characteristics of participants. Results were classified into five main themes and 11 sub-themes:

**Table 1:** Demographic characteristics of the participants in the FGDs

Variable	Frequency	Percentage
Age Groups		
30–34	4	14.2
35–39	13	46.4
40–44	6	21.4
45–49	5	18
Literacy Level		
Illiterate	0	0
Under High School Diploma	15	53.6
High School Diploma	6	21.4
Academic Degree	7	25
Job		
Housewife	19	68
Employed	9	32
Ethnicity		
Luri	13	46.4
Turk	2	7
Arab	8	28.6
Fars	5	18

1. Necessity of sexual health education for adolescent girls
  Different views were raised about the necessity of sexual health education for adolescent girls. Some participants believed that such education is necessary, others did not, and a third group considered the provision of such education only under particular conditions. The views on this issue were further classified into three following sub-groups. Although, there was no differences in participants which made them more or less likely to respond in any of the subthemes.
  1-1- Sexual health education is unnecessary for adolescent girls.
    These participants believed that their daughters will learn about sexual health matters through life and practice, and therefore targeted education is not necessary. They also stated that sexual health education encourages children to be rude. As stated by a 44-year-old mother, *“I do not agree that my daughter knows nothing about sex. So, I don’t answer her question because it makes them rude.”*
  1-2- Sexual health education is somewhat necessary.
    Most participants disagreed with the need for comprehensive sexual education girls. Instead, they believed that information provided by mothers should be limited to menstruation and puberty. A public-school principal shared the following opinion: *“Some students have a high level of understanding and intelligence, and we are not worried about training them. But, providing such trainings for some student may encourage them to experience such activities in reality.”* A school counselor pointed out the following*: “It depends on the student. Some of them must be provided with such information, and others must not receive such information in order to prevent his mind from digression.”*
  1-3- Sexual health education is an important necessity.
    Some participants agreed that the current generation is different from previous and they need to have knowledge about everything. A 37-year-old mother shared her thoughts: *“Girls need to know everything. We had a fanatic family and received no training in sexual issues from our mother. But, nowadays the relationships of boys and girls are quite different, and girls may be quickly and easily be deceived by boys. So, at least they have to be careful not to be deceived, receive emotional trauma, or get pregnant.”*
  2- Sources of sexual health education for adolescent girls
    Participants expressed different views regarding the appropriate sources for sexual health information.
  2-1- Mothers
    Most of the mothers agreed that education can be provided by parents at home. This was felt to be the preferred method because children are more likely to obey their parents at young ages. No opposition to this view was raised by the other respondents.
  2-2- School teachers and counselors
    Some mothers believed that teachers and counselors are the best persons to provide sexual health education. They pointed out that teachers and counselors could educate the girls about the sexual issues. As stated by a 33-year-old mother, *“I do not want my daughter give information from wrong reasons, so much better if the counselor says.”*
  2-3- Health educators
    Some participants regarded health educators and health care workers as best positioned to provide sexual education training because they have more knowledge on these issues.
  3- Barriers to providing sexual health education to adolescent girls
    Most participants believed that major barriers prevent the education of adolescent girls regarding sexual health issues, but their individual views differed. The barriers identified by the respondents were classified as follows:
  3-1- Shame and embarrassment surrounding discussions of sexual health
    A 40-year-old mother said, *“I am ashamed to say something about such issues to my daughter. My daughter also does not ask me because she does not feel comfortable with me.”* A mother who is a midwifery expert working in a health center stated the following: *“I try to answer many questions in school, but I feel embarrassed to say anything about such issues to my 13-year-old daughter. I see the changes in her behaviors and signs of puberty, but I don’t how to react to her questions.”*
  3-2- Fear of increasing temptation among adolescent girls
    Some participants believed that knowing about sexual health may cause adolescent girls to be curious or tempt them to engage in risky sexual behaviors. They assumed that if their daughters learnt about the sexual matters, and their mothers spoke about sex and sexuality, they had been persuaded to have sex relationships. A 33-year-old mother with a 15-year-old girl shared her perspective as: *“If girls permit to know about contraception, they may do anything wrong out of their family sight. If they know what to do, nothing bad may happen and the family may never notice.”*
  3-3- Lack of necessary skills for communicating with adolescents
    Some mothers pointed out they lack necessary skills for communicating with their adolescent girls, and considered that as a significant obstacle to the provision of sexual education. A middle-aged mother said*:“My daughter needs to trust me and ask me about the sexuality issues. But, I don’t know how I can listen to her, and speak about her concerns.”* Almost all participants in group discussions agreed with this idea and accepted this skill deficiency. In interviews with the key informants, this finding was confirmed too.
  4- Sources of information for knowledge on sexual issues
    Another important subject was how mothers derive information regarding sexual health.
  4-1- Information sought by oneself
    Some participants stated that they learned about sexual health by themselves and that they did not need help from anyone. An Arab 33-year-old mother said, *“When I got married, I did not know anything, and my wedding night was my life’s worst memory. Hence, I am always worried about my daughter. I wish she does not get married.”* One of the school counselors stated the following: *“My mother was very shy and she had told me nothing about menstruation, so I had many problems. It is very difficult to me to talk to my daughter about these issues. I hope she will be trained on these things.”*
  4-2- Information provided by relatives or friends
    A few of mothers stated that girls receive minimal information about sexual issues from sisters and cousins. Some of them indicated that they had not received enough information, and therefore could not play the role of an educator.
  4-3- Information provided by teachers in schools
    A few of the mothers reported that teachers provided limited information about menstruation and other sexual health issues. A mother said, *“My daughter says that in her school, if she wants to ask her question about sexuality issues, teachers just tell about menstruation and not more. Meanwhile, the information which is provided by theses teachers is limited.”*
2. Recommendations for providing sexual health education to adolescent girls
  Participants shared useful and practical recommendations for training mothers to provide sexual education. Most respondents agreed on the importance of training mothers to effectively communicate with their adolescent daughters and empower them to address questions about sexual health. Some of them mentioned necessity of improving communication skills, aside from sexual health education. A mother who is a general practitioner said, *“I understand the scientific issues, but I need to be trained on how to communicate with my children and how to explain sexual issues to them. Communication skills are important, even in relation to our husbands and sons.”* Some participants stated that mothers must know about sex-related issues, such as puberty-related changes, health during puberty, menstruation, sexually transmitted diseases (e.g., AIDS and hepatitis), risky behaviors, reproductive system infections, sexual dysfunction, contraception, unplanned pregnancy, relationships with the opposite sex, and good communication.

## Discussion

Most participants believe that sexual health education for adolescent girls must be initiated by mothers at home; nevertheless, some opposed the provision of in-depth education altogether. Participants shared their fears regarding stimulating the curiosity of their children and the temptation to engage in sexual relations. These findings are consistent with a cross-sectional study on junior high students in Malaysia, which revealed that most girls agree that their mothers must be the first person to provide information on puberty and sexual issues [16]. A study conducted on junior high students in Lagos, Nigeria showed that most teachers deem training on sexual issues a responsibility of families and believe that the home is better than school for the transmission of such information [17]. In Abolqasemi et al.’s [12] qualitative study on the views of health educators in schools, most believed that sexual health education should come from parents.

In the study presented here, Iranian mothers identified some barriers to sexual health education. Insufficient knowledge about sexual issues, fear of temptation among girls to engage in sexual relations, and lack of necessary skills for communicating with adolescent girls, is all barriers to parents’ role as educators. Meanwhile, this study showed that discussion about sexual health is often described as embarrassing. The findings of Latif- Nejad et al.’s [18] qualitative study revealed that cultural resistance more effectively constrains the nature and content of sexual health education than do religious prohibitions. In a review conducted by Bahrami et al. [13], the religious beliefs and challenges that adolescents encounter in Iran present difficulties in communicating with parents regarding sexual health matters. In some other studies, the insufficient knowledge and information of mothers on puberty, their failure to inform their daughters, and embarrassment or discomfort in explaining these issues were reported as the main reasons sexual health information was not provided [11]. In a study carried out by Mirzaei [9], parents stated that providing sexual information to adolescents may reduce modesty and promote sexual promiscuity.

The mothers in this study stated that they cannot easily communicate with their children, and thus find discussing sexual health difficult. A study in Ethiopia showed that communication between parents and adolescents about sexual and reproductive health is insufficient; only about a third of the participating students had a discussion about such issues with their parents [15]. A 2013 study in Ireland indicated that although most parents claim that they are comfortable discussing sexual health with their children, few of them directly speak about contraception and condom use with their children [19]. A 2009 study in Nigeria showed that about one-quarter of parents believed that their children should not know about contraception and that exposure to such information may encourage them to experience sexual activities at an early age [17]. In research in Ireland, some parents expressed concern over the possibility that adolescents will be encouraged to experience sexual activities after receiving information on safe sex [19]. In Nigeria, 56% of the teachers participating in a study expressed a belief that sexual health education may promote early exposure to sexual relations [20].

Most participants in this study deemed it necessary to educate mothers, and about half of the mothers believed that they need communication training. Two of the mothers stated that such training is also required for fathers. The results of other studies in Iran and elsewhere emphasize the need to educate and empower families, especially mothers [10, 12, 18, 21]. Another important requirement is to ensure that the manner by which parents communicate and interact with their children and the training provided to families prepare them in a way that will enable them to answer their children’s sexual questions at any age [10]. Another study recommended that parents establish good and stable relationships with their children and discuss ethics and society’s values and norms with them [22].

The topic addressed in this study is taboo in the Iranian context, and some of the focus group participants did not want to talk about sexual health education. Additionally, the focus groups are not representative of an entire population, and the participants’ responses may have been influenced by others. Given the small non-randomized sample, the generalizability of the results should be approached with caution.

## Conclusion

Well-informed and prepared mothers are thought to be the best sources of sexual health education for adolescent girls; therefore, mothers should be trained for effective communication regarding sexual health issues. Developing appropriate sexual health programs for mothers of different ethnicities and cultures is important. A needs assessment should be conducted to develop a training program that is based on the needs of mothers. These programs should then be presented to education departments and used in parent training courses.
